# A specific diagnostic metabolome signature in adult IgA vasculitis

**DOI:** 10.1007/s11306-024-02107-0

**Published:** 2024-05-24

**Authors:** Alexandre Boissais, Hélène Blasco, Patrick Emond, Antoine Lefèvre, Adrien Bigot, Yanis Ramdani, Nicole Ferreira Maldent, Denis Mulleman, Evangéline Pillebout, François Maillot, Alexandra Audemard-Verger

**Affiliations:** 1 Department of Internal Medicine and Clinical Immunology, University Hospital Center of Tours, Tours, France; 2 Biochemistry and Molecular Biology Department, University Hospital Center of Tours, Tours, France; 3 UMR 1253, iBrain, University of Tours, 37000 InsermTours, France; 4 In Vitro Nuclear Medicine Department, University Hospital Center of Tours, Tours, France; 5 Center for Molecular Biophysics, UPR CNRS 4301, Tours, France; 6 Department of Rheumatology, University Hospital Center of Tours, Tours, France; 7 Department of Nephrology, Saint Louis Hospital, APHP, Paris, France; 8 Nanomedicines and Nanoprobes Department, University of Tours, Tours, France

**Keywords:** IgA vasculitis, Henoch-Schönlein purpura, Metabolomics, Diagnosis, Signature

## Abstract

**Introduction:**

IgA vasculitis diagnosis relies primarily on clinical features and is confirmed by pathological findings. To date, there is no reliable noninvasive diagnostic biomarker.

**Objective:**

We aimed to explore the baseline serum metabolome of adult patients with IgA vasculitis to identify potential diagnostic biomarkers.

**Methods:**

We performed a study comparing the serum metabolome of patients with IgA vasculitis to that of patients with inflammatory condition, namely spondyloarthritis. Serum analyses were performed by high-performance liquid chromatography-mass spectrometry.

**Results:**

Fifty-five patients with IgA vasculitis and 77 controls with spondyloarthritis (age- and sex-matched) were included in this study. The median age of IgA vasculitis patients was 53 years. Two-thirds of patients were female (n = 32). At the time of vasculitis diagnosis, 100% of patients had skin involvement and 69% presented with glomerulonephritis (n = 38). Joint and digestive involvement were observed in 56% (n = 31) and 42% (n = 23) of patients. Four discriminative metabolites between the two groups were identified: 1-methyladenosine, L-glutamic acid, serotonin, and thymidine. The multivariate model built from the serum metabolomes of patients with IgA vasculitis and spondyloarthritis revealed an accuracy > 90%. As this model was significant according to the permutation test (p < 0.01), independent validation showed an excellent predictive value of the test set: sensitivity 98%; specificity 98%, positive predictive value 97% and negative predictive value 98%.

**Conclusion:**

To our knowledge, this study is the first to use the metabolomic approach for diagnostic purposes in adult IgA vasculitis, highlighting a specific diagnostic metabolome signature.

**Supplementary Information:**

The online version contains supplementary material available at 10.1007/s11306-024-02107-0.

## Introduction

Immunoglobulin A vasculitis (IgAV) is induced by deposits of immune complexes mostly containing IgA-1 in small vessels, leading to purpura, arthralgia, abdominal pain and/or glomerulonephritis. IgAV is more frequent during childhood and has a good prognosis (Gardner-Medwin et al., 2002). In adults, the disease is more scarce but remains a real challenge for physicians due to a worse prognosis with the occurrence in some patients of chronic glomerulonephritis (García-Porrúa et al., 2002; Pillebout et al., 2002; Watts et al., 2005; Yang et al., 2005), and acute digestive involvement with a risk of perforation (Audemard‐Verger & Terrier 2017; Ebert, 2008; Tracy et al., 2019).

IgAV is known to occur following infection and is referred to as a “seasonal” disease demonstrated by an epidemiological peak in autumn and winter in children and spring and summer in adults (García-Porrúa et al., 2002). In most patients, a virus, bacteria or a drug is a trigger leading to the onset of vasculitis (Brandy-García et al., 2020; Endo et al., 2019; Itoh et al., 2020; Mazumder et al., 2020). In adults, IgAV diagnosis is based upon clinical symptoms and laboratory parameters as well as the analysis of a histological sample, e.g., skin or kidney sample which may show IgA deposits. However, in approximately 20% of cases, IgA deposits are absent from skin biopsies (Ozen et al., 2019). In case of renal involvement, a renal biopsy can be performed to establish the diagnosis (J. A. Mills et al., 1990). This is an invasive procedure with a significant risk of bleeding (Poggio et al., 2020). Moreover, analyses by the department of pathology require approximately 15 days; this is not negligible considering that a certain diagnosis determines the treatment when the clinical form is immediately severe. Therefore, the identification of both non-invasive and robust diagnostic biomarkers is lacking.

Metabolomics is based on hybrid technologies to explore the metabolome defined as small molecules, in order to provide a metabolic signature. This approach is nowadays used to identify specific metabolic profiles for both diagnostic and prognostic biomarkers and also evaluate therapeutic effectiveness. So far, in the field of IgAV, metabolomic studies have been overlooked, most studies focusing on the pivotal role of the immune system. However, inflammation is known to disrupt metabolic pathways and reciprocally metabolic imbalance can modify immune system homeostasis. Here, we aim to explore the plasma metabolome of adult IgAV patients to identify potential diagnostic biomarkers.

## Materials & methods

### Patients and controls

This is a *post-hoc* study. The samples analyzed were sera from adult IgAV patients enrolled in the “HSPrognosis” cohort (Berthelot et al., 2018). The local ethical committee of Assistance Publique–Hôpitaux de Paris (AP-HP, approval number 10.649bis) approved this study, and written informed consent was obtained from all patients. Inclusion criteria for both studies were: (1) age > 18 years, (2) IgA vasculitis, and (3) diagnosis of IgAV between January 1990 and January 2015. Patients were considered to have IgAV if they presented with (1) purpura, (2) histologically proven small vessel vasculitis, (3) histologically confirmed IgA deposits and (4) involvement of at least one organ among the kidney, joints, or intestinal tract. Patients were excluded if there was no skin purpura, if they had been treated with immunosuppressive drugs or glucocorticosteroids (GS) within 2 weeks before the onset of a skin rash, if they had thrombocytopenia and if they did not consent and/or were under the age of 18 years old. We compared IgA vasculitis to patients with inflammatory condition without vasculitis, namely spondyloarthritis (SpA). The aim was to find a specific metabolomic signature of the disease and not of its inflammatory nature. The two groups were age- and sex-matched. Sera were taken from the COMARIS1 cohort including patients with axial spondyloarthritis. Patients were included between March 2013 and October 2014 within the HUGO network (Hôpitaux Universitaires du Grand Ouest—Western France University Hospitals). Patients gave their written informed consent to be in the study. Patients had to be at least 18 years of age and fulfill the Assessment of Spondylo-Arthritis International Society criteria for axial SpA (Hounoum et al., 2015). Patients had to be eligible for a TNFi in accordance with the French marketing authorization of pharmaceutical products. Patients must not have received methotrexate during the previous 3 months and were not allowed to participate in the study if they had previously received adalimumab, or if they have received more than one TNFi. The study was performed in accordance with the ethical standards of the Declaration of Helsinki and was approved by the Institutional Review Board. Patient consent was obtained. Controls were excluded if they have been treated with GS, DMARDs and/or biologics.

### Study design

We first aimed to identify a diagnostic model for IgA vasculitis. Thus, we studied and compared the serum metabolome of patients with IgA vasculitis and controls including spondyloarthritis (SpA) patients, using a metabolomic approach.

The secondary objective of this study was to highlight the metabolomics profile of IgA vasculitis according to the various clinical phenotypes (endophenotypes). Patients were separated according to their organ involvements: skin, joint, digestive or renal damages (all patients had skin involvement).

### Metabolic analysis

Sera were analyzed by High-Performance Liquid Chromatography coupled to mass spectrometry, as described in the literature (Hounoum et al., 2015), after extraction with 100 µl of methanol from 20 µl of serum. An UPLC Ultimate WPS-3000 system coupled to a Q-Exactive mass spectrometer, operated in positive and negative electrospray ionization modes (analysis for each ionization mode), was used for this analysis.

Liquid chromatography was performed using a Phenomenex Kinetex 1.7 μm XB—C18 column (100 mm × 2.10 mm) maintained at 40 °C. Two mobile phase gradients (preceded by an equilibrium time of 3 min); A: H_2_O + 0.1% formic acid; B: methanol + 0.1% formic acid were used. The gradient was maintained at a flow rate of 0.4 mL/min. The multistep gradient was programmed as follows: 0–2 min, 0.1% B; 2–6 min, 0.1–25% B; 6–10 min, 25 − 80% B; 10 − 12 min, 80–90% B; 12–16.5 min, 90–99.9% B; 16.5–20 min, 99.9–0.1% B. Two different columns were used to increase the metabolic coverage. Accordingly, a hydrophilic interaction liquid chromatography (HILIC) column (CORTECS UPLC HILIC 150 mm × 2.1 mm × 1.6 μm (WATERS)) was also used. Two mobile phase gradients were also used (A: H_2_O + 10 mM formiate NH4 + 0.5% formic acid; B: acetonitrile + 10 mM formiate NH_4_ + 0.5% formic acid) and the gradient was maintained at a flow rate of 0.3 mL/min. The multistep gradient was programmed as follows: 0–1.5 min, 95% B; 1.5–8 min, 95–82% B; 8–15 min, 82–75% B; 15–15.5 min, 75–25% B; 15.5–16 min, 25–3% B; 16–22 min, 3–95% B. The injection volume was 5 μL. During full-scan acquisition, which ranged from 58 to 870 m/z, the instrument operated at 70,000 resolution (m/z = 200).

As required for all biological analyses, the pre-analytical and analytical steps of the experiment were validated by findings of Quality Control (QC) samples, prepared from a mix of 20 random samples from our cohort extracted using the same process as for patients. Consequently, they represented both technical and extraction variations. Coefficients of variation [CV% = (the standard deviation/ mean) × 100], were calculated from all metabolite data. Metabolites with a CV in QCs > 30% were excluded from the final dataset. QCs were analyzed at the beginning of the run, every 10 samples and at the end of the run.

Targeted analysis was applied to the samples, based on a library of standard compounds (Mass Spectroscopy Metabolite Library (MSML) of standards, IROA Technologies). The following criteria were followed to identify the metabolites: (1) retention time of the detected metabolite within ± 20 s of the standard reference, (2) exact measured molecular mass of the metabolite within a range of 10 ppm around the known molecular mass of the reference compound, and (3) correspondence between isotopic ratios of the metabolite and the standard reference. The signal value was calculated using Xcalibur™ software (Thermo Fisher Scientific, San Jose, CA) by integrating the chromatographic peak area corresponding to the selected metabolite. At this step, the dataset contained the identity of the metabolites and the corresponding area for all samples after analysis and validation by a mass spectrometry specialist. Data were normalized to the sum, log-transformed and auto-scaled before statistical analysis.

### Statistical analyses

**Fig. 1:**
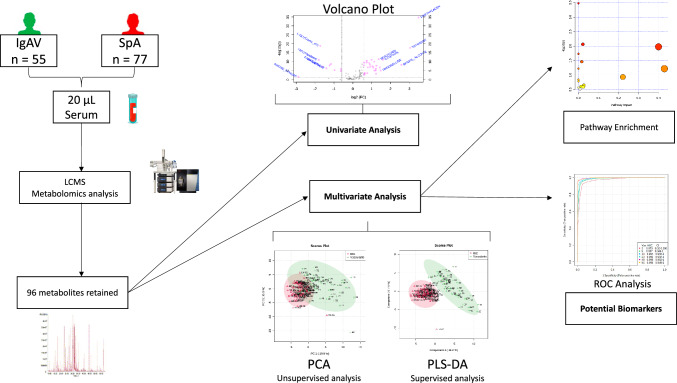
Study design and data analysis workflow

The study design and data analysis workflow are illustrated in Fig. 1.

#### Univariate analysis

The 2 groups were matched with the online statistical software: Medistica.pvalue.io (Available on: https://www.pvalue.io/fr).

The analysis of qualitative variables was performed by a Chi-squared test and the comparison of quantitative variables was performed by the Welsh two-sample t-test.

Univariate analysis was represented by the volcano plot based on the Fold Change (FC) value and the significance level based on the Wilcoxon non-parametric test via the free software Metaboanalyst version 5.0 (https://www.metaboanalyst.ca/MetaboAnalyst/ModuleView.xhtml). To highlight the most discriminating parameters, we used the False Discovery Rate. We highlighted only metabolites with a Flow Change > 0.5 or < −0.5 and an adjusted p-value < 0.1

#### Multivariate analysis

Metabolome profiles were also analyzed by a multivariate approach to identify a diagnostic prediction model as described previously. Samples were classified by an unsupervised multivariate descriptive analysis called Principal Component Analysis (PCA) to determine the distribution of the samples and possible outsiders, but also by a supervised analysis based on Partial Least-Squares Discriminant Analysis (PLS-DA). The score plot shows the distribution of the classified samples. The value of Variable Influence on Projection (VIP) represents the importance of the metabolites according to the PLS-DA supervised model. To test the relevance of both the highlighted metabolites and the model, accuracy evaluation and the permutation test were performed. To test the pertinence and the quality of the model, we used two independent cohorts to establish independent validity of the diagnostic model. For this purpose, we randomly divided our database into a training set comprising thirty-six patients from the IgAV group and fifty-one from the spondyloarthritis group.

The remaining samples constituted the “test set”, including one third of the IgA vasculitis samples and one third of the samples with SpA. The analysis was performed blind for the diagnosis.

Receiver Operating Characteristic (ROC) curves were generated by Monte Carlo cross validation (MCCV) using balanced sub-sampling. In each MCCV, 2/3 of the samples were used to identify the most important metabolites and were then used in the classification model to infer the diagnosis of the remaining 1/3 of the samples. The classification method was PLS-DA as previously defined. We generated different models according to the supervised multivariate analysis PLS-DA. From the training set, we selected the model with the higher area under the curve, being significant and we only kept the 15 most significant metabolites. We then predicted the diagnosis of patients from the test set. We independently repeated this process ten times to estimate the average sensitivity, specificity, positive and negative predictive value in the test set.

### Metabolic pathway analysis

Metabolic pathways and enrichment analyses were systematically performed with the five most discriminant metabolites highlighted by PLS-DA model. Metabolic pathway enrichment and pathway topology analysis were conducted using MetaboAnalyst version 5.0 (https://www.metaboanalyst.ca/) to determine a probability for each metabolic pathway identifying its relevance.

The value of pathway impact was calculated as the sum of importance measures of the metabolites, normalized by the sum of importance measures of all metabolites in each pathway. Metabolic pathways are represented as a network of chemical compounds with metabolites as nodes and reactions as edges.

## Results

### Patients

Fifty-five sera from patients with IgAV and 77 controls with SpA were included in the analyses. The main clinical and biological characteristics of IgAV patients, along with age- and sex-matched SpA patients, are shown in Table 1. Briefly, at baseline, all IgAV patients presented skin involvement, 69% presented renal involvement (n = 38), 56% joint involvement (n = 31), and 42% had gastro intestinal involvement (n = 23). SpA controls were age- and sex-matched as shown in Table 1.

**Table 1:** Baseline characteristics of IgA vasculitis and spondyloarthritis and clinical characteristics of patients with IgA vasculitis

	IgA Vasculitis (n = 55)	Spondyloarthritis (n = 77)	p
*Sex*			
Female, n (%)	32 (58%)	37 (48%)	0.25
*Age*			
Median, years, Q1-Q3	53.0 [30.0; 61.0]	42.0 [35.0; 51.0]	0.19
*Clinical involvement*			
Skin	55 (100%)	–	–
Renal	38 (69%)	–	–
Joints	31 (56%)	–	–
Gastro-Intestinal	23 (42%)	–	–

### Metabolomic profiles

**Fig. 2:**
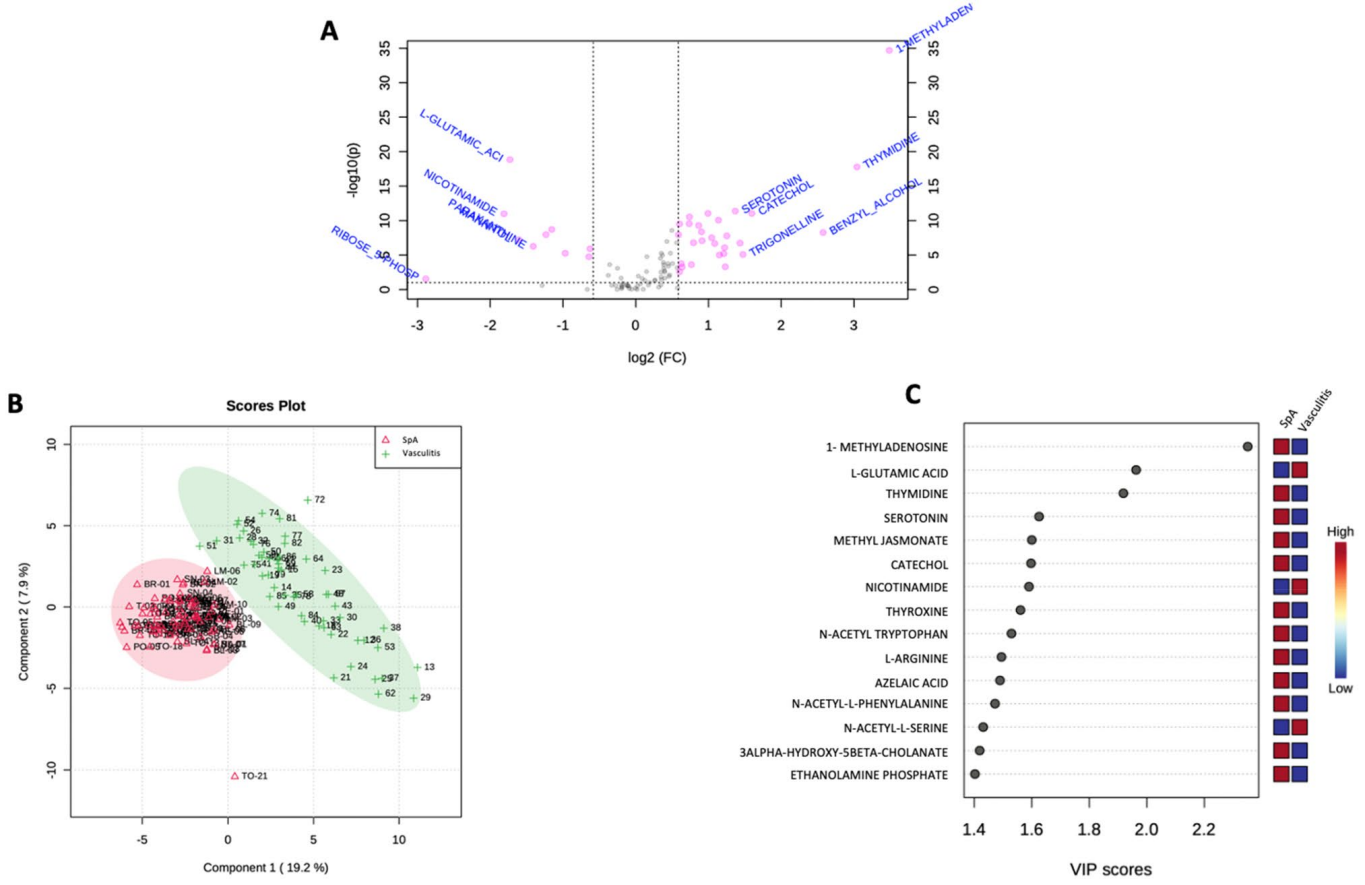
**A** Univariate analyses via volcano plot based on fold change and adjusted p-value, highlighting 38 metabolites. The x-axis represents the Fold Change between groups (log scale) while the y-axis represents adjusted p-value obtained by a t-test of the difference in metabolite concentrations (negative log scale). **B** Score scatter plot based on PLS-DA models to explain the diagnosis (green cross for patients with IgA vasculitis and red triangle for patients with spondyloarthritis). **C** Classification of the top 15 of metabolites highlighted with PLS-DA model according to the VIP score on the x-axis. The colored boxes on the side of the figure represent the relative concentration for each metabolite in each group (a red box indicates an increase in the relevant concentration of the metabolite, whereas a blue box indicates a decrease in the concentration of the metabolite)

Volcano plot analysis (Fig. 2A) highlighted a significant difference in the concentrations of 38 metabolites (of the 96 metabolites measured) between IgAV and SpA, with an adjusted p-value < 0.05. The 10 most discriminant metabolites were 1-methyladenosine (adjusted p-value 208.10^−35^, FC = 11.195), L-glutamic acid (adjusted p-value 144.10^−19^, FC =  − 0.30), Thymidine (adjusted p-value 164.10^−18^, FC = 8.23), Serotonin (adjusted p-value 421.10^−12^, FC = 2.58), Catechol (adjusted p-value 901.10^−12^, FC = 3.02), Methyl Jasmonate (adjusted p-value 901.10^−12^, FC = 1.99), Nicotinamide (adjusted p-value 103.10^−11^, FC =  − 0.28), Thyroxine (adjusted p-value 293.10^−11^, FC = 1.67), N-acetyl-tryptophan (adjusted p-value 86.10^−11^, FC = 2.2) and L-Arginine (adjusted p-value 279.10^−10^, FC = 1.66). The whole set of 38 metabolites highlighted by the univariate volcano plot are reported in Appendix 1.

The unsupervised analysis (PCA) did not find any outliers among the samples. The multivariate analysis supervised by PLS-DA split the samples into 2 distinct groups as shown in the score plot (Fig. 2B). Model performance was robust, with a predictivity test of 95% and a significant permutation test (p < 0.01) highlighting its robustness. The important features contributing to this model are shown in Fig. 2C. The set of metabolites shown in the graph representing the most important features based on PLS-DA analysis were identical to those found by univariate analysis. The 3 metabolites with a VIP score > 1.8 were also the first 3 metabolites identified by univariate analysis: 1-methyladenosine, L-glutamic acid and Thymidine. The metabolic pathway analysis from the discriminating metabolites highlighted by the PLS-DA model did not reveal any significant metabolic pathway (Appendix 2).

### The metabolomics profile allows the diagnosis to be predicted in an independent population

As the multivariate model was significant to predict the diagnosis of IgAV on the entire cohort, we applied the same strategy previously described to test the performance of diagnosis prediction in an independent cohort.

The PLS DA diagnosis models built from the metabolome of patients from the 10 independent training sets showed excellent performance. Accordingly, the ROC curves based on the PLS-DA model from several features showed excellent criteria such as an AUC at 0.988 (p < 0.01) based on a model from less than 10 features.

**Fig. 3:**
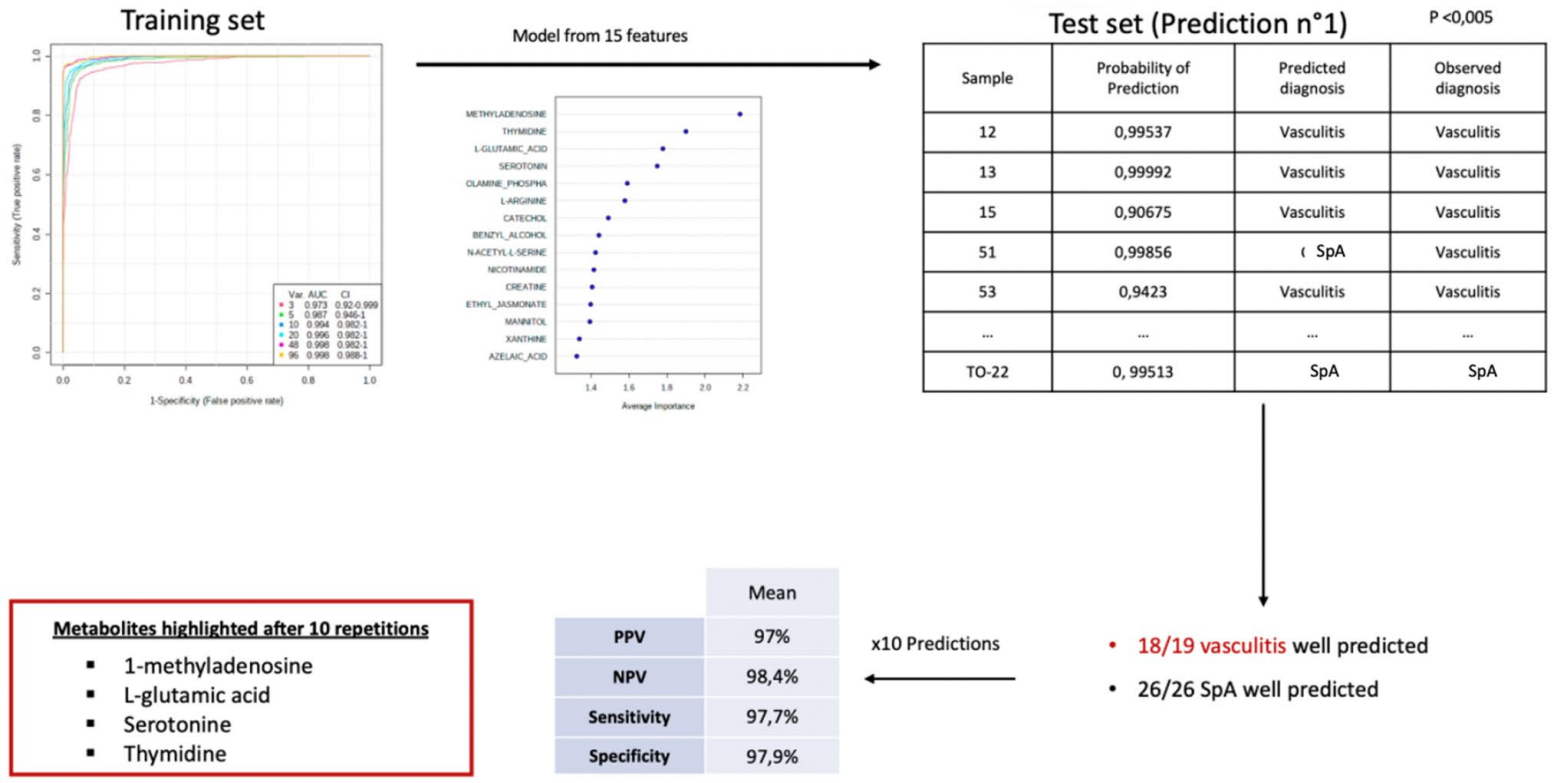
Prediction of IgA vasculitis in an independent cohort from the plasma metabolome profile of patients from the training set. ROC curves, obtained from PLS-DA models, allowed the diagnosis prediction to be compared to the observed diagnosis

Moreover, the performance of the PLS DA diagnosis models of patients in the test sets was also excellent. Figure 3 shows an example of prediction, as previously described, to predict the diagnosis in the test set, showing a ROC curve with AUC at 0.994 (p < 0.01). Overall, we obtained a very good reproducibility with similar predictive values, i.e. sensitivity, specificity, PPV and NPV > 97% in the test set.

Using Venn diagrams, 4 metabolites stood out because there were present in the 10 independent predictions (among the multivariate and univariate analyses performed): 1-methyladenosine, L-glutamic acid, Serotonin and Thymidine.

### The metabolomics profile does not allow the determination of the endophenotype of patients with IgA vasculitis

We also investigated whether metabolome analysis of IgA vasculitis patients could distinguish the different clinical phenotypes (endophenotypes) according to the organ involved. For this purpose, we grouped patients according to organ involvement (skin, renal, joint or gastrointestinal).

We then conducted statistical analyses of the metabolomics profiles as previously described, comparing the different endophenotypes 2 by 2 for more clarity and comparability. The PLS-DA model did not allow us to split significatively, in each group, IgA vasculitis and skin involvement versus patients with IgA vasculitis with skin and joint involvement (Appendix 3). The PLS-DA model was able to separate these 2 groups, despite a poor accuracy, with a non-significant permutation test (p = 0.68).

Thus, the metabolomic profile did not appear to discriminate between the different endophenotypes within IgA vasculitis patients. Therefore, metabolic pathway analysis was not performed.

## Discussion

So far, many studies have focused on the pivotal role of the immune system during the course of adult IgAV. To our knowledge, the present study is the first to use a metabolomic approach for diagnostic purposes in IgAV. Our main result was the identification of a baseline “metabolic signature” in IgAV patients.

Through this approach using a robust statistical method**,** we were able to build a diagnostic model that highlights 4 metabolites. From these data, we suggest that such a metabolomic signature could be used as a diagnostic biomarker in daily practice. Indeed, both positive and negative predictive values were > 97% in the prediction of IgAV diagnosis.

Obviously, these results need to be confirmed in an independent cohort including other forms of small-vessel vasculitis such as cryoglobulinemia and ANCA vasculitis in order to clarify whether the metabolomic signature that we described here is specific to IgAV or not. This latest point is crucial to assist clinicians in the diagnosis of IgAV, especially in the 20% of patients in whom IgA deposits are lacking. Besides the traditional tissue-based disease paradigm, the metabolomic approach could also improve our knowledge of vasculitis as a whole, for further therapeutic developments.

Metabolomics is a powerful tool for exploring the metabolic imbalance. Metabolomics represents a potential tool to identify either diagnostic or prognostic biomarkers in human disease. The study of metabolomics for several rheumatic diseases, including rheumatoid arthritis, lupus, osteoarthritis and vasculitis, has revealed distinctive metabolic signatures (Ding & Mohan, 2016; Geetha et al., 2022; Kim et al., 2014; Zhai et al., 2010). It should be noted that none of these studies was able to successfully build a diagnostic model. For instance, in the field of vasculitis, a study of Takayasu arteritis revealed a distinct baseline signature with decreased HDL, glucogenic amino acids and increased choline, LDL and N‐acetyl glycoprotein levels (Guleria et al., 2015).

Regarding the role of the 4 metabolites identified in our study (1-methyladenosine, glutamic, serotonin, and thymidine), it is interesting to note that these metabolites are not interconnected and do not reflect the engagement of a single metabolic pathway. Moreover, unlike other metabolites such as pyruvate, lactate, choline or malonate, none of these metabolites are known to play crucial roles in shaping the immune system (Mehta et al., 2010; E. L. Mills et al. 2016; Palsson-McDermott & O’Neill, 2013). Altogether, it suggests that these metabolites are not involved in the pathology of IgAV but are rather a consequence of immune disturbance.

As previously mentioned, using metabolomics in adult IgAV appears to be a new and innovative approach. A Turkish study used a non-targeted metabolomics approach to search for prognostic biomarkers of occurrence of renal damage in an IgAV pediatric population (Demir et al., 2020). Of the 45 patients, 6 developed renal involvement at 6 months during the follow up. The authors suggested that DHAP, prostaglandin D2/I2, porphobilinogen, 5-methyltetrahydrofolic acid, and N-Acetyl-4-O-acetylneuraminic acid/N-Acetyl-7-O-acetylneuraminic acid may serve as biomarkers for predicting kidney disease. These results were not in concordance with our data that failed to show any specific profiles of patients with renal involvement. Such a difference may be explained in the first place by the differences between the studies (pediatric population versus adult population), but also because the analyses performed did not measure the same metabolites as our panel.

Our work has several strengths. It is a preliminary work that has identified a metabolomic signature. To our knowledge, this is the only study using metabolomics for diagnostic purposes in adult IgA vasculitis. To minimize the confounding factors that may affect the metabolomic differences, we included age- and sex-matched controls. Indeed, among the different factors that may influence systemic metabolism, age and sex are well-known parameters (Chaleckis et al., 2016; Krumsiek et al., 2015). Moreover, rather than healthy controls, we enrolled patients suffering from inflammatory disease without vasculitis such as axial SpA. This option was intended to highlight a specific IgAV profile rather than a metabolomic signature of autoimmune disease or inflammation. Finally, as this is a rare disease (more frequent in adults than in children), the number of patients included is substantial.

There are also some limitations to this work. As mentioned above, this work needs to be confirmed by an independent cohort including other forms of small-vessel vasculitis such as cryoglobulinemia and ANCA vasculitis in order to clarify whether the metabolomic signature that we described here is specific to IgAV or not.

Our study has exemplified the power of metabolomics for identifying potential diagnostic metabolic biomarkers. If validated in separate cohorts, these biomarkers may have the potential to diagnose IgAV avoiding the need to perform invasive biopsies, which could have a deleterious effect. Such a novel approach may have a profound impact on the clinical assessment and management of IgA vasculitis.

## Conclusion

To our knowledge, this study is the first to use the metabolomic approach for diagnostic purposes in adult IgA vasculitis, highlighting a specific diagnostic metabolome signature. This metabolic signature needs to be confirmed in an independent cohort including controls suffering from other forms of vasculitis if it is to be used as a reliable diagnostic biomarker.

### Supplementary Information

Below is the link to the electronic supplementary material.

Supplementary file1 (DOCX 1354 KB)

## Data Availability

Original data can be available by contacting Dr AUDEMARD-VERGER alexandra.audemardverger@univ-tours.fr.

## Abbreviations

IgAV: IgA vasculitis
GS: Glucocorticosteroids
SpA: Spondyloarthritis
DMARDs: Disease-modifying antirheumatic drugs
