# Supplementary material for: A specific diagnostic metabolome signature in adult IgA vasculitis
Source: Metabolomics. 2024 May 24;20(3):61. doi: 10.1007/s11306-024-02107-0 (PMC11126474; doi:10.1007/s11306-024-02107-0)
Supplement: Supplementary file 1 — Supplementary file1 (DOCX 1354 KB) [file 11306_2024_2107_MOESM1_ESM.docx]

Appendix 1: List of the 38 metabolites highlighted via univariate analysis (Cf. Figure 1A representing the volcano plot)


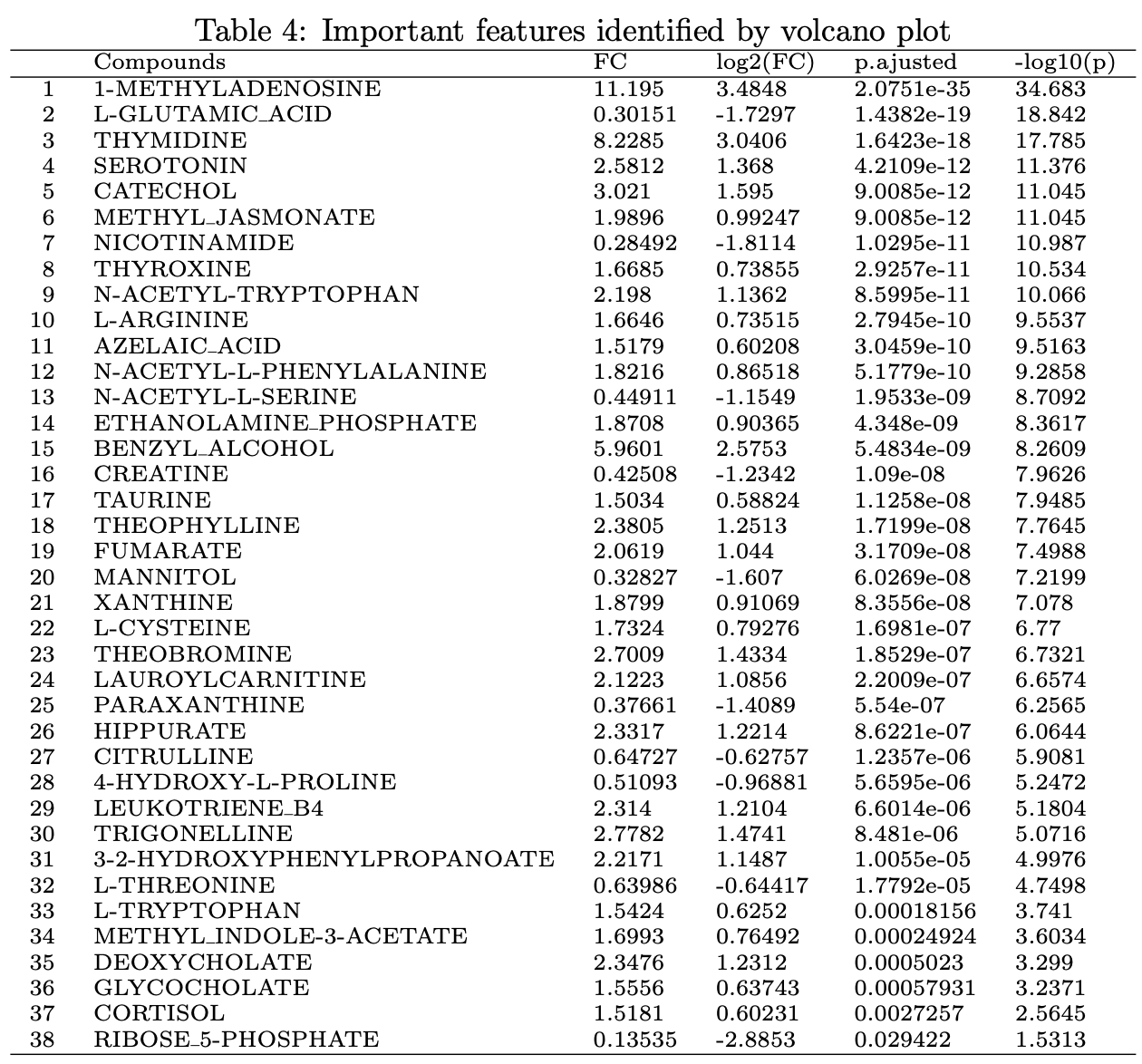


Appendix 2: Metabolic pathway analyses of the 15 discriminating metabolites highlighted by the PLS-DA model. No significant metabolic pathway is highlighted.


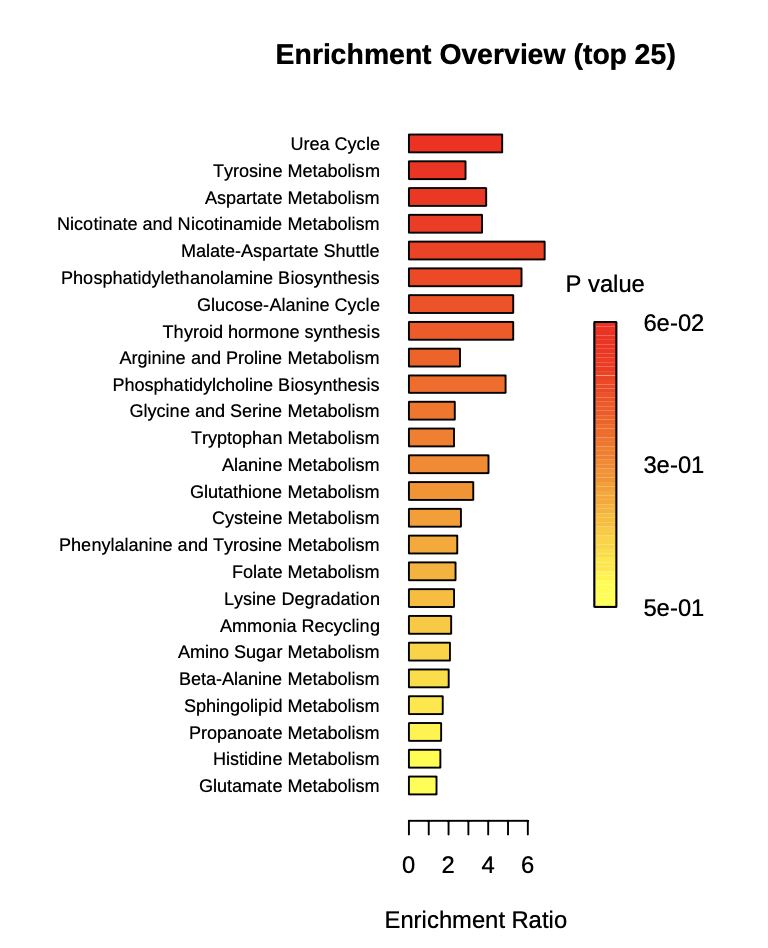


Appendix 3**:** Multivariate analysis of the serum metabolome profile of IgA vasculitis with skin involvement (C) and IgA vasculitis with skin and articular involvement (CA).

1. Score scatter plot based on PCA analysis of patients according to the organ involved
2. Score scatter plot based on PLS-DA models to explain endophenotypes in IgA vasculitis (green cross for patients with IgA vasculitis with skin and articular involvement and red triangle for patients with IgA vasculitis with skin involvement only).
3. Prediction accuracy is not significant as with the Permutation test. To conclude, this model does not allow the separation of endophenotypes.
4. Classification of the top 15 metabolites highlighted with the PLS-DA model according to the VIP score on the x-axis. The colored boxes on the side of the figure represent the relative concentration for each metabolite in each group (a red box means an increase in relevant concentration of the metabolite, whereas a blue box means a decrease in the concentration of the metabolite).

**A**

**C**

**B**


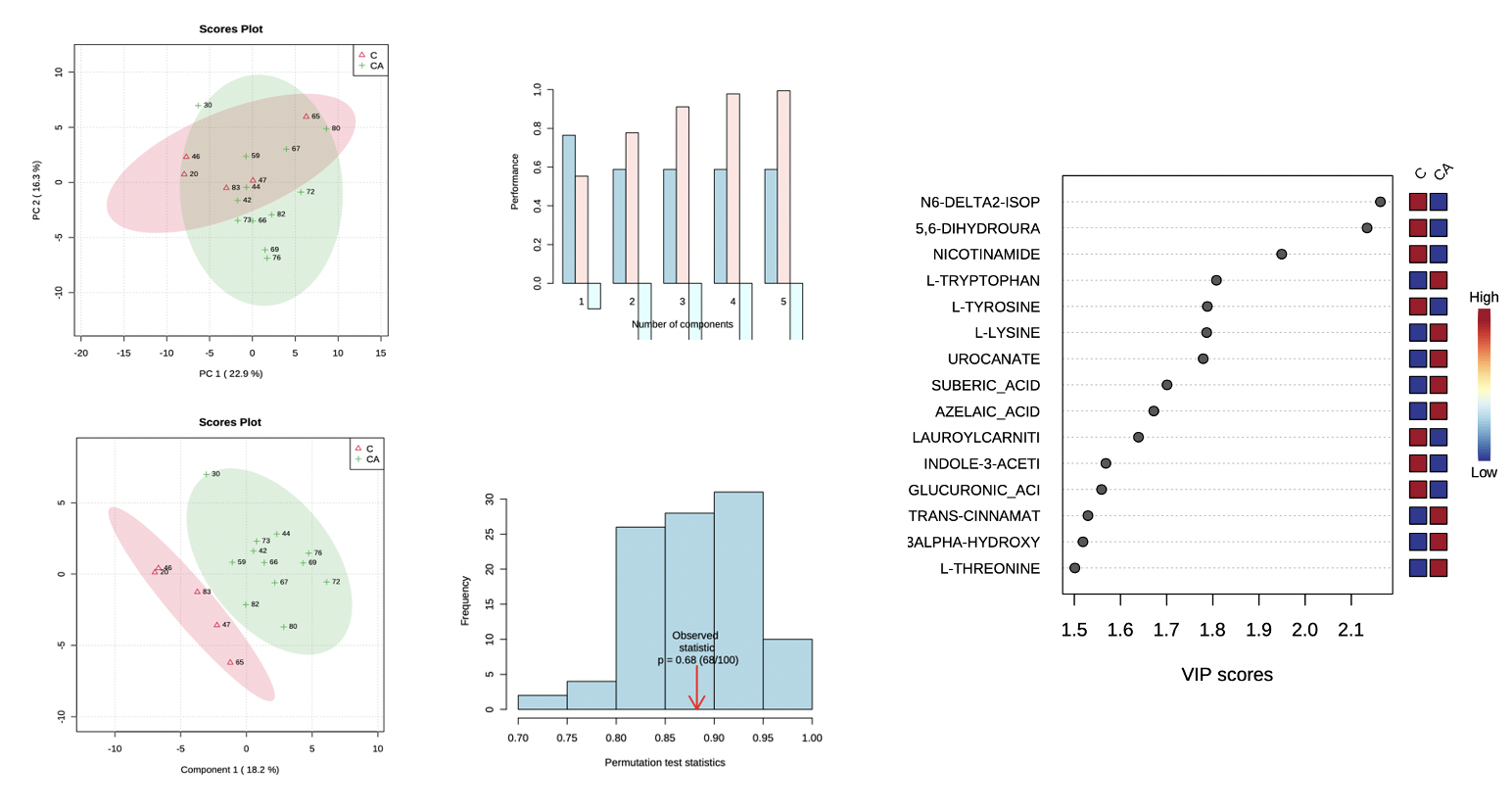

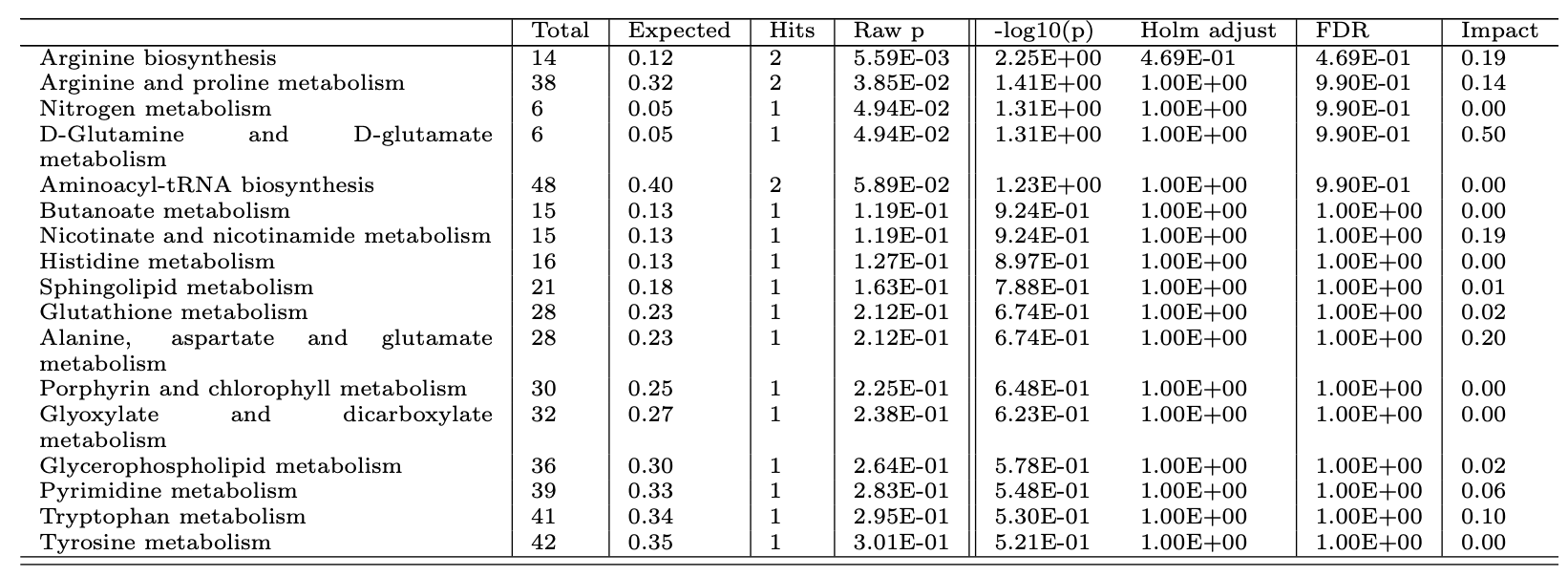


**D**
